# Supplementary material for: PKCδ deficiency inhibits fetal development and is associated with heart elastic fiber hyperplasia and lung inflammation in adult PKCδ knockout mice
Source: PLoS One. 2021 Jul 1;16(7):e0253912. doi: 10.1371/journal.pone.0253912 (PMC8248728; doi:10.1371/journal.pone.0253912)

Fig.2B Original data-1
(Gel: 0.6% Agarose Gel stained with EtBr)

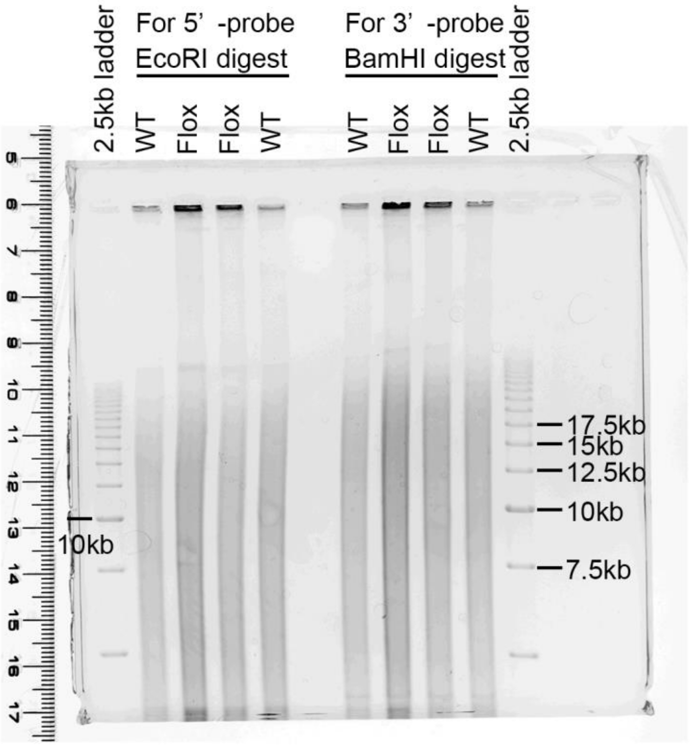

Fig.2B Original data-2 (Blot: DIG)

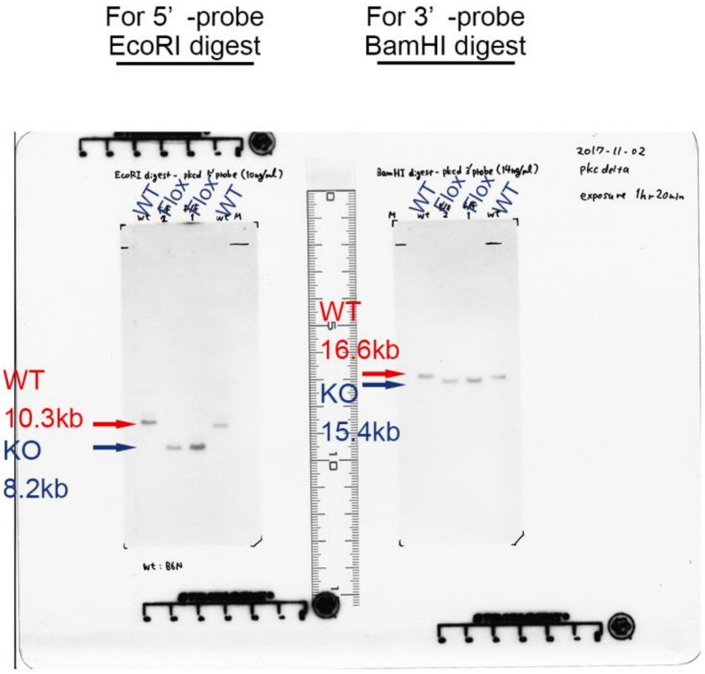

Fig.2C Original data-1
(1% Agarose Gel)

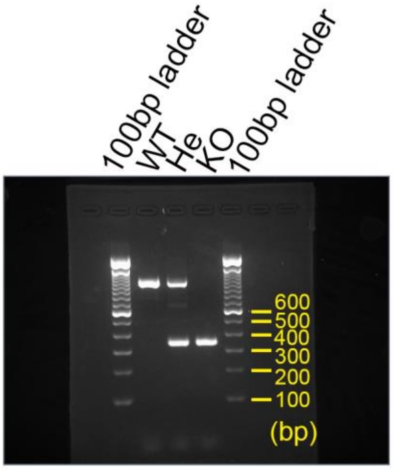

Supplement: S1 Raw images — (PDF) [file pone.0253912.s001.pdf]
