# Supplementary material for: PKCδ deficiency inhibits fetal development and is associated with heart elastic fiber hyperplasia and lung inflammation in adult PKCδ knockout mice
Source: PLoS One. 2021 Jul 1;16(7):e0253912. doi: 10.1371/journal.pone.0253912 (PMC8248728; doi:10.1371/journal.pone.0253912)
Supplement: Attachment — Submitted filename: Responses_Letter.docx [file pone.0253912.s003.docx]

**Response to Editor Comments**

Please review your reference list to ensure that it is complete and correct. If you have cited papers that have been retracted, please include the rationale for doing so in the manuscript text, or remove these references and replace them with relevant current references. Any changes to the reference list should be mentioned in the rebuttal letter that accompanies your revised manuscript. If you need to cite a retracted article, indicate the article's retracted status in the References list and also include a citation and full reference for the retraction notice.
Response: We checked the reference list of our paper. No literature was retracted.

Some problems with statistics and interpretations still remain. In addition, a few minor points need to be addressed.

**Response to Reviewer's Comments**

Reviewer #1: The efforts to include Kaplan Meier analysis and some statistical analysis is appreciated.

Response: We thank the reviewer for reading our revised manuscript in detail and helping us improve our paper. We have read your opinion carefully. Please find our response below.

Comment 1: 332- 339: this section is confusingly written and I think needs clarification. Are the authors saying the embryos were implanted at Mendelian ratios but that most KOs were dead at E11.5?

Response: In lines 303-323, we described that the proportion of the number of WT, He, and KO fetal sacs at E11.5 followed Mendelian laws. However, the proportion of surviving KO embryos (with heartbeat) was significantly lower than WT and He at E11.5. We apologize if this was not conveyed appropriately. We have changed the table (Table 3 in the revised manuscript) to include data showing the number of implantations and the survival rate. Additionally, we suggested that this phenomenon might occur due to a problem with KO development in the mother's body.

Comment 2: Table 2 still appears to lack stats. The data in this table are none-the-less interesting but the interpretation remains difficult. Het off spring die as readily as KO offspring from the KO crosses suggesting a rearing issue rather than a viability issue for the young. Drawing conclusions, clearly they KOs can produce offspring but are unable to rear the young. This is difficult to research and draw solid conclusions from which the authors acknowledge.

Response: We thank the reviewer for appreciating the data from Table 2. We agree with the reviewer that these data suggest possible childcare issues. Since Table 2 does not have enough data to analyze and draw statistically significant conclusions, we have removed Table 2 and its description from the revised manuscript.

Reviewer #2: The manuscript has been revised and some of my earlier points answered. The following points should be addressed:
Response: We thank the reviewer for reading our revised manuscript in detail and helping us improve our paper. We have read your opinion carefully. Please find our response below.

Comment 1. Lines 236-240 are not very conclusive or informative. These should be rephrased or deleted. The discussion of this point on line 424 may suffice.
Response: We thank the reviewer for the comment. Since Table 2 did not have enough data to analyze and draw statistically significant conclusions, we have removed Table 2 and its description from the revised manuscript.

Comment 2. Is there experimental justification to use the term hypertrophy on line 460 (see replies to previous comments)?

Response: We could not provide additional experimental data showing that the enlarged heart in a PKCδ KO mouse corresponds to cardiac hypertrophy. Therefore, we deleted the word “hypertrophy” at all relevant instances and instead rewrote all statements signifying that the heart of the PKCδ KO mouse was "enlarged".

Reviewer #3: I have no other comments. All my questions and suggestions were successfully responded by authors.

Response: We thank the reviewer for reading our treatise in detail and for the constructive feedback.
